# Post-vaccine glomerulonephritis in an infant with hereditary C2 complement deficiency: case study

**DOI:** 10.3325/cmj.2013.54.569

**Published:** 2013-12

**Authors:** Tanja Kersnik Levart

**Affiliations:** Department of Pediatric Nephrology, University Medical Centre, Ljubljana, Slovenia

## Abstract

We describe a case of a post vaccine immune complex-mediated glomerulonephritis in an infant with compound heterozygous mutations of C2 complement component gene, which is the first such case in the literature. The three and a half months old boy presented with clinical and laboratory signs of nephritic syndrome and was successfully treated with methylprednisolone. An explanation of such a clinical picture may lie in the interaction between C2 deficiency and vaccination.

Nephritic syndrome may occur at any age, but is an uncommon finding in infants (1-6). It is caused by proliferative changes and inflammation in the glomeruli – proliferative glomerulonephritis (GN), which can be a primary/isolated disease or a consequence of a systemic disease. The most common types of primary proliferative GN in children are post-infectious GN, IgA nephropathy, and membranoproliferative GN, and the most common types of proliferative GN due to a systemic disease are Henoch Schönlein GN and lupus GN (1,5,6). All these conditions are immunologically mediated with in situ immune-complex formation or passive immune-complex trapping in the glomeruli and activation of secondary immune mechanisms like complement system.

We describe a case of presumably post-vaccine immune-complex mediated GN in a three and a half months old boy in whom two heterozygous mutations on a C2 complement component gene were found. The possible adverse event after vaccination was reported to the National Institute of Public Health.

## Case report

The patient was born to healthy, unrelated Caucasian parents, at 39 weeks of gestation, weighing 4160 g. The pregnancy was uneventful. His three and a half years old sister had a history of allergy to eggs and had been treated for many otitis media infections. The mother’s second pregnancy ended with spontaneous abortion in the first trimester. The mother had a microhematuria of unknown cause. Patient’s parents gave a written informed consent for writing of this case report.

### Patient’s history and physical status

The boy was evaluated at the age of six weeks due to rash and suspected allergy to milk. The rash subsided when the mother, who was breastfeeding him, went on a milk-free diet. He was otherwise healthy until the day after his first vaccination against diphtheria, tetanus, pertussis, poliomyelitis, Haemophilus influenza type B, and Pneumococcus when he was three months old. The first vaccine was a combined one, containing active substances of Diphtheria toxoid, Tetanus toxoid, Bordetella pertussis antigens (Toxoid, Filamentoushaemagglutinin), type 1 poliomyelitisvirus (inactivated), type 2 poliomyelitisvirus (inactivated), type 3 poliomyelitisvirus (inactivated), polysaccharide of *Haemophilus influenzae* type b conjugated to the tetanus protein and other ingredients including saccharose, trometamol, aluminum hydroxide, Hanks’ medium without phenol red, acetic acid, and/or sodium hydroxide for pH adjustment, formaldehyde, phenoxyethanol, and water for injections. The second vaccine was a Pneumococcal polysaccharide conjugate vaccine containing Pneumococcal polysaccharide serotype 1^1,2^, 4^1,2^, 5^1,2^, 6B^1,2^, 7F^1,2^, 9V^1,2^, 14^1,2^, 18C^1,3^, 19F^1,4^, and 23F^1,2^ (^1^adsorbed on aluminum phosphate, ^2^conjugated to protein D, derived from non-typeable *Haemophilus influenzae* carrier protein, ^3^conjugated to tetanus toxoid carrier protein, ^4^conjugated to diphtheria toxoid carrier protein). Other excipients in the second vaccine were sodium chloride and water for injections.

The boy was febrile for three days starting from the day after vaccination, was well again for another fourteen days, and then suddenly presented with macrohematuria and mild periorbital edema. He was sent for evaluation to our department. The physician did not see him when was febrile, so there were no firm data regarding the cause of the fever. Theoretically, this may have been the case of infection or nonspecific fever after vaccination.

At admission, he looked well although somewhat irritated, with no signs of respiratory tract infection, no skin rash, and with mild periorbital edema. He was very well grown, with weight of 7900 g (81st percentile) and height of 68 cm (95th percentile). The blood pressure was normal – 92/55 mm Hg, with pulse around 138/min. Physical exam of his heart, lung, abdomen, and extremities showed normal results. Peripheral pulses were palpable.

### Course of treatment and medical examinations

We confirmed macrohematuria, accompanied also by nephrotic range proteinuria (maximum value of u-protein/creatinine was 4104 g/mol), mild hypoproteinemia (minimal value of serum proteins 47 g/L), hypoalbuminemia (minimal value of serum albumin 30 g/L), and elevated serum concentration of urea (maximum 10.9 mmol/L) and creatinine (maximum 66 µmol/L – estimated glomerular filtration rate 41 mL/min/1.73 m^2^). Immunoglobulin levels were as follows: IgE 13 kU/L (reference range from 0 to 13 kU/L), IgG 5.72, IgA 0.52, and IgM 2.0 g/L (reference range for IgG 2.41 to 6.13, IgA 0.1 to 0.46, IgM 0.26 to 0.6 g/L), while antistreptolysin 0 level was 54 IU/mL (reference range from 0 to 170 IU/mL). Blood pressure was normal all the time, but the boy needed furosemide to maintain diuresis. Kidneys were of normal shape and size but hyperechoic on ultrasound. At presentation, total hemolytic complement activity was very low (alternative pathway 40 IU; reference range from 40 to 120 IU, classical pathway 9%; reference range from 72 to 128%). C3 component was only mildly decreased (851 mg/L; reference range from 970 to 1576 mg/L), while concentrations of all other complement components were within the reference range, except for C2 component, which was extremely low (1.72 mg/L; reference range from 14 to 25 mg/L). C5-9 lytic complex was elevated in urine (424 µg/L; reference value less than 30 µg/L) and in plasma it was within the reference range (303 µg/L; reference range 300-350 µg/L). Anti-extractable nuclear antigen, antinuclear antibodies, anti-double stranded DNA antibodies, anticardiolipin antibodies, anti-beta2-glycoprotein I antibodies, antibodies against C1q, and anti-neutrophil cytoplasmic antibodies were negative.

On the basis of clinical examination and the results of basic laboratory and morphologic tests, we reasonably suspected that the boy had nephritic syndrome, which was probably post-vaccine and that something was wrong with his complement system (very low C2).

Renal biopsy showed a diffuse, nonuniform endoproliferative (20/25 = 80%), mesangioproliferative (5/25 = 20%), and extracapillary crescentic (6/25 = 24%) GN. Extensive focal (40%-50%) active mixcellular tubulointerstitial nephritis was also found. Immunofluorescence analysis showed IgA stained +/++ (on a scale from 0 to 4+), IgG +-, IgM +, kappa and lambda +/++, C3 +/++, C1q +-, and fibrin/fibrinogen +-. Deposits, mostly IgA and C3, were located in the glomerular capillary wall and in the mesangium. Electron microscopy showed that deposits were mesangial and sub epithelial in cases when they did not form characteristic humps. It was concluded that this was an immune complex-mediated GN, most probably an atypical post-infectious GN (post-vaccine GN) with predominant IgA and C3 deposits in immune-complexes and no humps on electron microscopy. The composition of immune deposits described in our patient could also be found in IgA GN or Henoch-Schönlein Purpura, but the clinical course of our patient excluded these two conditions with great probability.

Due to unavailability of corresponding examinations, we could not prove specific antigens in glomerular immune complexes, but on the 25th day after vaccination it was confirmed that the boy had formed specific antibodies against some vaccines (IgG against Haemophilus influenza *type B* was 0.5 mg/L, protective titer 1.0 mg/L; IgG against Pneumococcus 8.4 mg/L, protective titer 30.0 mg/L; IgG against tetanus 0.27 IE/mL, protective titer 0.15 IE/L; IgG against diphtheria 0.12 IE/L, protective titer 0.1 IE/L). IgG against pertussis and poliomyelitis could not be tested in our laboratory. The boy was not re-vaccinated until the age of one.

The patient was treated with three pulses of methylprednisolone (10 mg/kg) for three consecutive days, followed by 1 mg/kg of methylprednisolone daily for 6 weeks. Thereafter, the steroids were tapered gradually and withdrawn completely in a 4-month period. He went into complete remission after 6 weeks of treatment and at the moment of writing of this report had been off all treatment for 4 months and was doing fine. He had normal renal function, normal blood pressure, no proteinuria or hematuria, normal kidney ultrasound, but had persistently decreased total hemolytic complement activity (alternative pathway 41 IU; reference range 40-120 IU, classical pathway 7%; reference range 72%-128%). C3 component and C5-9 lytic complex in urine normalized, while C2 component stayed low (3.16 mg/L; reference range 14-25 mg/L). The patient was found to be compound heterozygous for C2 gene. Two heterozygous mutations were found on his gene for C2 component. The first one was 28-base pair deletion of genomic DNA, which results in a premature termination of transcription. The second was a missense mutation that changes glutamate into aspartate at the position 298. The same mutations were found also in his older sister, while the parents refused to be tested.

## Discussion

This report presents a case of a three and a half months old patient who developed nephritic syndrome, which is an uncommon finding in infants. The patient had post-vaccine immune-complex mediated GN and two heterozygous mutations on C2 complement component gene.

Genetically determined C2 deficiency is the most common of inherited complement deficiencies. Homozygous or complete C2 deficiency can present with a variety of symptoms – from asymptomatic state to systemic lupus erythematosus-like illness, polymyositis, glomerulonephritis, Hodgkin lymphoma, vasculitis, Henoch-Schönlein purpura, and recurrent pyogenic infections with encapsulated bacteria, such as Streptococcus pneumoniae, Haemophilus influenza type b, and Neisseria meningitides. Patients may also have a combination of multiple autoimmune phenomena, especially various cutaneous manifestations and pyogenic infections (7-9). On the other hand, in most individuals partial C2 deficiency has no clinical importance (7,10,11).

Development of GN after vaccination has been reported earlier but as a very rare event and never in a patient with C2 deficiency. Post vaccine GN can present with a variety of clinical and histological pictures. It has been described after hyperimmunization with pertussis vaccine as a diffuse vasculitis and death (12), after influenza H1N1 vaccination as a membranous GN (13), after hepatitis B vaccine as a vaccine-related systemic lupus erythematosus (14), and after pneumococcal vaccination as a crescentic GN due to anti glomerular basement membrane disease (15). Nephrotic syndrome has been described after measles vaccination (16) and after anti rabies vaccine (17).

Our patient was simultaneously vaccinated against diphtheria, tetanus, pertussis, polio, Haemophilus influenza type B, and Pneumococcus. Eighteen days after vaccination he presented with clinical and laboratory signs of nephritic syndrome. Immune complex-mediated GN was histologically confirmed with predominant IgA and C3 deposits in immune complexes and no humps on electron microscopy. Specific antigens in glomerular immune complexes could not have been proven, but it was proven that the boy had formed specific antibodies in protective titers against all the received vaccines. Therefore, the predisposition for his atypical post-infectious or post-vaccine GN theoretically could have been any compound of any vaccine, as well as an infection he could have had before the onset of nephritic syndrome. In addition, two heterozygous mutations on C2 complement component gene were found, both already described (18,19), but not in compound heterozygous patients, which could explain the extreme C2 deficiency in our patient.

There was a question about patient’s further vaccination. Since approximately 50% of C2-deficient patients have an increased susceptibility to blood borne infections caused with encapsulated organisms (eg, pneumococcus, H. influenza, and meningococcus), vaccination represents a treatment of choice against sepsis, meningitis, arthritis, and osteomyelitis (7-9). On the other hand, they are at increased risk of developing systemic lupus erythematosus, glomerulonephritis, inflammatory bowel disease, dermatomyositis, anaphylactoid purpura, and vasculitis (7-9), all these conditions being possibly triggered by environmental factors like infection or vaccination. The boy’s parents were very anxious about further vaccination, so it was decided against it and the child was put on antibiotic prophylaxis with penicillin until a different decision is made.

In conclusion, this is the first case report that describes post vaccine immune complex-mediated GN in an infant with compound heterozygous mutations on C2 complement component gene. An explanation of such a clinical picture may lie in the interaction between C2 deficiency and vaccination.
